# Ligand-free all-inorganic metal halide nanocubes for fast, ultra-sensitive and self-powered ozone sensors†

**DOI:** 10.1039/c9na00219g

**Published:** 2019-05-22

**Authors:** K. Brintakis, E. Gagaoudakis, A. Kostopoulou, V. Faka, A. Argyrou, V. Binas, G. Kiriakidis, E. Stratakis

**Affiliations:** Institute of Electronic Structure & Laser (IESL), Foundation for Research and Technology (FORTH) Hellas P.O. Box 1385 Heraklion 70013 Crete Greece akosto@iesl.forth.gr stratak@iesl.forth.gr; University of Crete, Department of Physics 710 03 Heraklion Crete Greece; University of Crete, Department of Materials Science and Technology 710 03 Heraklion Crete Greece; Crete Center for Quantum Complexity and Nanotechnology, Department of Physics, University of Crete 71003 Heraklion Greece

## Abstract

Ligand-free all-inorganic lead halide nanocubes have been investigated as ozone sensing materials operating at room temperature. It is found that the nanocubes, crystallined in the orthorhombic CsPbBr_3_ structure, can operate at room temperature, be self-powered and exhibit high sensitivity and remarkable repeatability. More importantly, they demonstrate higher sensitivity (54% in 187 ppb) and faster response and recovery times compared to hybrid lead mixed halide perovskite (CH_3_NH_3_PbI_3−*x*_Cl_*x*_) layers, which is the only lead halide perovskite material tested for ozone sensing, to date. Following the exposure to an ozone environment, the stoichiometry and the morphology of the nanocubes remain unaltered. The facile and easy fabrication process together with the high responsivity and stability to the ozone environment makes the bare CsPbBr_3_ nanocubes a promising material for sensing applications. The sensing properties of the nanoparticulate metal halides presented here provide new exciting opportunities towards engineering reliable and cheap sensing elements for room-temperature operated and self-powered sensors.

## Abstract 2

Quick and cost-effective fabrication of metal-halide nanostructure ozone sensors.

## Graphical abstract

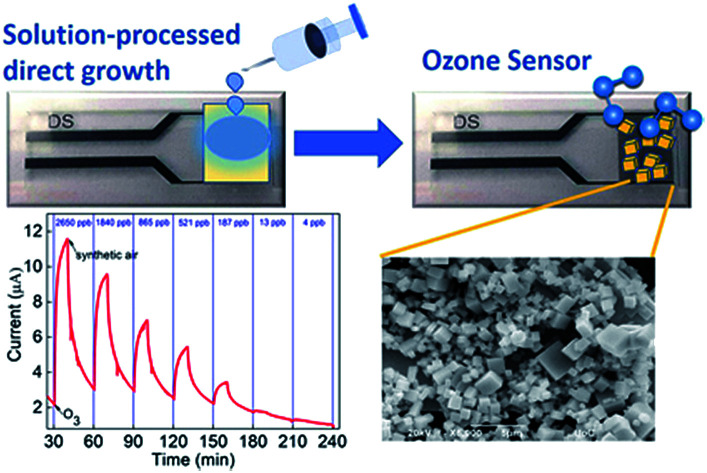

## A. Introduction

Perovskites are the materials with the formula ABX_3_, where a cation ‘A’ occupies the corner positions and a cation ‘B’ the center of the unit cell, while an anion ‘X’ is located at the unit cell faces.^1^ The material family of perovskites includes several oxides, as well as metal halides. The compositional/structural features or the physical properties (optical and electrical) of those materials have been found to change upon an external stimulus such as humidity variations, gas exposure or temperature difference.^2,3^ Owing to this property, perovskite oxides have been successfully tested as sensing elements to detect analytes including humidity,^4,5^ gases^6–23^ and temperature changes.^5^ More recently, metal halide perovskites, which demonstrate attractive opto-electronic properties for photovoltaic applications,^24^ have also been tested as sensing materials. In this case, reversible variations of their fluorescence, phosphorescence or chemical resistivity upon applying an external stimulus due to intercalation processes, surface absorption, trap passivation or ion exchange have been reported and used for temperature,^25^ humidity,^26–28^ gas^29–36^ and metal ion^37–39^ sensing.

At the same time, the intense research interest for developing new sensors featuring high sensitivity, high selectivity, fast response and long-term stability leads to test, nanostructured materials as sensing elements. The structuring of the matter at the nanoscale offers a high surface-to-volume ratio, which favours the adsorption of gases on the sensing material due to higher interaction between the analyte and the sensing part.^35^ Despite the large variety of colloidal synthesis procedures for nanomaterials of high crystallinity quality and finely tuned size, shape, and composition,^40–45^ only a few examples have been reported for sensing applications by using such materials. Among them, nanostructured metal halide materials have been used for sensing metal ions (copper or mercury),^37,39^ gases (hydrochloric acid or oxygen)^33,34^ and humidity.^26^

According to our knowledge there is no sensor based on metal halide nanostructures for ozone gas sensing. The ozone is detected both in the Earth's upper atmosphere and at the ground level. Ground level ozone which is called “bad ozone” is produced from the reaction between nitrogen oxides and hydrocarbons, when pollutants are emitted by cars, chemical solvents and industrial releases.^46^ Ozone is highly toxic when its concentration in the air exceeds 1 ppm. Breathing ozone can trigger a variety of health problems including chest pain, coughing, throat irritation, and congestion.^47^ It can worsen bronchitis, emphysema, and asthma and can also reduce lung function and inflame the linings of the lungs. Repeated exposure may permanently scar lung tissue. Ozone at concentrations higher than 50 ppm presents risks to life, and an ozone concentration of 1000 ppm or more, causes death in a short time.^48^ Thus, ozone sensors are crucial for our modern life well-being, providing us information for health protection.

Ozone sensors can be classified according to the property/feature used for sensing as optical,^49^ optochemical,^50^ electrochemical^51^ and conductometric^52^ and the sensing materials in these cases are metal oxides such as In_2_O_3_, SnO_2_, WO_3_, TiO_2_, CuAlO_2_, and SmFeO_3_ in the film form.^53^ The responsiveness and the sensitivity of ozone sensors in these cases can be affected by metal oxide synthesis/film deposition through factors, such as film thickness, film porosity and grain size.^53^ These ozone sensors operate at temperatures well above room temperature, their sensitivity is limited to a few hundreds of ppb and they are not self-powered devices, as they require to be switched on before their operation by an external stimuli like UltraViolet (UV) irradiation or heating. In this context, there is an increasing demand for self-powered and ultra-sensitive down to ppb ozone sensing systems.

Recently, an organic–inorganic metal halide film fabricated by a spin coating procedure has been used as a self-powered, ultrasensitive and room-temperature operating ozone sensing material.^30^ This material can detect ozone concentrations down to a few ppb with a response time of 200 seconds; at the same time, no external stimulus is required to initiate the sensing process. However, the complex and time-consuming fabrication (spin coating and subsequent annealing in an inert atmosphere), together with the short sensor lifetime, due to film degradation under ambient conditions and the reaction with ozone, challenges the scientific community to discover more stable metal halides to be used for this application.

The present work is the first to demonstrate the application, of all-inorganic lead halide nanocrystals as ozone sensing materials with superior operational stability. In particular, well-crystalline and stable under ambient conditions CsPbBr_3_ nanocubes have been directly grown onto a substrate *via* a solution-based precipitation method, in a few seconds. It was found that the high active surface area of the nanocubes, together with the enhanced interaction between the ozone analyte and the sensing part, touching directly the electrical contacts, gives rise to high sensitivity and responsiveness with remarkable stability. Indeed, it is shown that the response time is two times lower, compared to that measured for organic–inorganic metal halide films.^30^ At the same time and contrary to organic–inorganic layers, the response effect is completely reversible. It is concluded that the proposed system, which combines a simple and quick fabrication process with high sensitivity and long-term stability under ambient conditions, is promising for ozone sensing applications.

## B. Results and discussion

### Fabrication of the ozone sensor

**Fig. 1:**
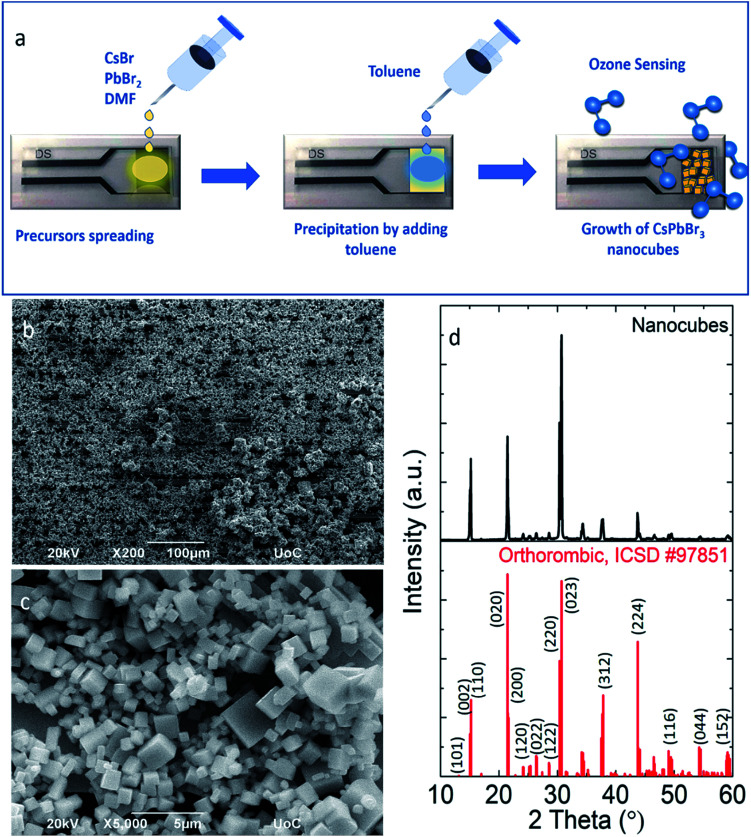
Schematic representation of the ozone sensor fabrication based on CsPbBr_3_ nanocubes (a), SEM images (b and c) and X-ray diffraction pattern ((d) black curve) of the nanocubes prior to ozone treatment. A reference pattern of the orthorhombic (ICSD, #97851) crystal structure of CsPbBr_3_ is provided for comparison ((d) red curve).

All-inorganic lead halide perovskite nanocubes with sizes ranging from 500 nm to 1 μm have been fabricated directly on a pre-patterned electrode substrate through a simple and fast re-precipitation method at room temperature. Particularly, a small volume of an already prepared precursor solution is deposited on the substrate and then an equal amount of toluene is spread over the whole substrate area (Fig. 1a). Following this process, ligand-free nanocubes, well-defined in shape, have been directly grown onto the substrate (Fig. 1b and c). Similarly, re-precipitation solution-based methods have been used to synthesize different morphologies of metal halide nanocrystals ranging from spheres,^54–56^ cubes^43^ and platelets^57–59^ to more anisotropic morphologies such as nanowires^45,58^ and nanorods.^58,60^ Moreover, ligand-free nanocubes grown on substrates by spin coating of the precursor solution, followed by heat treatment,^61^ have been used as efficient materials for nanolasers.^62^ X-ray diffraction analysis revealed the high-crystallinity of nanocubes, which is compatible with the orthorhombic structure of CsPbBr_3_ (ICSD, #97851). At the same time the X-ray diffraction spectra show no indication of a secondary phase. The stoichiometry is also confirmed by EDS analysis (Fig. S1a†) and the calculated energy gap (2.27 eV,) is very close to the reported band gap of the direct single crystal semiconductor CsPbBr_3_.^63^

### Sensing properties of the metal halide nanocubes

**Fig. 2:**
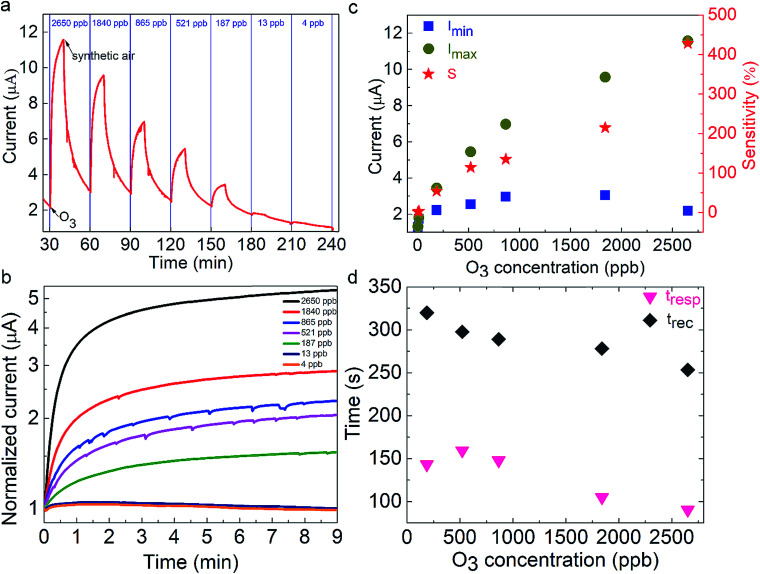
Electrical response of the all-inorganic perovskite nanocubes as sensing materials upon applying various ozone concentrations from 2650 down to 4 ppb as a function of the ozone exposure time (a and b). Sensitivity (*S*) and response (*t*_res_) and recovery time (*t*_rec_) as a function of gas concentration of the nanocube-based sensor (c and d).

**Fig. 3:**
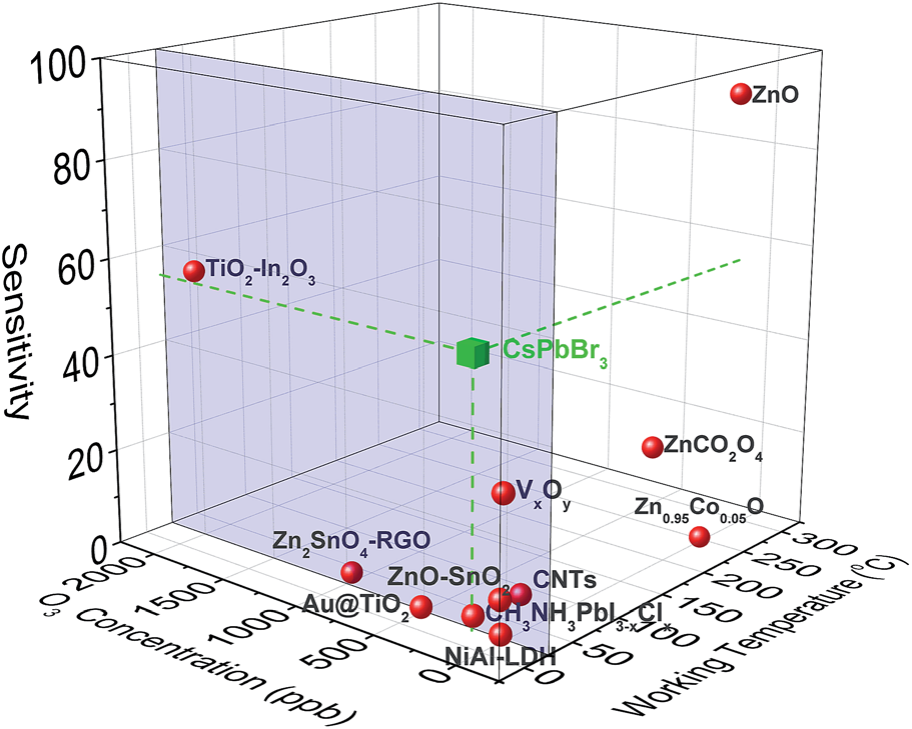
Sensitivity *vs.* O_3_ concentration and working temperature for the sensing elements found in the literature. The blue frame indicates the room temperature regime.

The nanocube-based sensor is placed into a homemade gas-testing chamber, while the ozone flow is digitally controlled by using a dual cell photometer. The most important parameters to characterize the performance of a sensor are the sensitivity, the response and recovery times, as well as the sensing reversibility. The initial current is measured to be approximately 2 μA (Fig. 2a). This high initial current value is due to the direct contact between the nanoparticulate sensing material and the platinum contacts, as well as the absence of organic ligands around the particles, which could behave as an insulating medium. CsPbBr_3_ perovskites absorb in the range of 400–500 nm depending on their shape and size, which lies in the visible range, thus explaining the initial current, when the sensor is exposed to ambient light.^31,42^ This current is two times higher than that of the hybrid organic–inorganic perovskite film (CH_3_NH_3_PbI_3−*x*_Cl_*x*_) deposited on the same electrode substrate and tested for a similar ozone sensing application.^30^ It has to be noted that no additional external stimulus is required for the sensor to be switched on to the initial state prior to ozone exposure. This is important in the case of using the sensor in a portable device. The sensing process is initiated by introduction a well-defined ozone flow of 500 sccm (standard cm^3^ min^−1^) into the chamber, controlled by using a flowmeter, giving rise to an electrical current increase. The sensor recovered to its original state upon introducing synthetic air in the chamber for 20 min with a similar flow. During the exposure and the recovery processes, the pressure in the chamber was kept constant and equal to 400 mbar. All the experiments were performed at room temperature. Fig. 2a presents the electrical response of the sensor, showing the immediate current increase upon exposure to ozone gas and the respective recovery of the response, within a few seconds after the ozone gas replacement with synthetic air. The same behaviour is observed for all tested ozone concentrations ranging from 2650 down to 4 ppb. The time in which the sensor can detect ozone is less than 1 min, indicating its fast response (Fig. 2b). Fig. 2c shows the detailed evolution of the maximum and the minimum current values, *I*_max_ and *I*_min_, as a function of the respective ozone concentrations tested. It can be observed that *I*_max_ increases upon increasing the ozone concentration, which is not the case for the minimum current value, which is always close to the starting value. The corresponding material sensitivity, calculated from the ratio [(*I*_max_ − *I*_min_)/*I*_min_] × 100% (Fig. 2c, red stars) is increased from 1.3% for 4 ppb to almost 428% for 2650 ppb ozone concentration. The 187 ppb corresponds to the ozone level that the sensor can measure with certainty, with 54% sensitivity. The position of our sensing element among the other reported semiconducting materials for ozone sensing^30,64–73^ is illustrated in Fig. 3. It can be observed that the nanocubes we report here exhibit the highest sensitivity amongst the other competitors under room temperature working conditions and detect the lowest ozone concentrations.

Two important parameters that determine the sensor quality are the response (*t*_res_) and the recovery (*t*_rec_) times. The former is defined as the time taken by the electrical current to reach 90% of its maximum value under an ozone flow, while the latter is the time taken by the current to become 10% of its maximum value, upon ozone removal. Fig. 2d presents the evolution of *t*_res_ and *t*_rec_ with the ozone concentration. Notably, *t*_res_ values lie between 100 s and 150 s while *t*_rec_ is between 250 s and 320 s. These values are almost two times higher than those measured for hybrid metal-halide thin films. Furthermore, the response time is almost unchanged for concentrations below 865 ppb and decreases at higher ones. In contrast, the recovery time decreases upon increasing the concentration. These values are close to those reported in the literature for O_3_ sensing materials (Fig. S2 and Table S1†). In particular, the response time of the nanocubes reported here is faster compared to that of hybrid organic–inorganic metal halides used as sensing elements and in the same order as that of oxides. Significantly faster response times were observed for diphasic sensing elements (NiAl-LDH,^65^ TiO_2_–In_2_O_3_,^67^ ZnO–SnO_2_ (ref. 68) and Au@TiO_2_ (ref. 64)). However, these materials are synthesized with more complex and time-consuming fabrication processes compared to the solution-processed direct growth proposed in this work.

### Short-term stability of the sensor

**Fig. 4:**
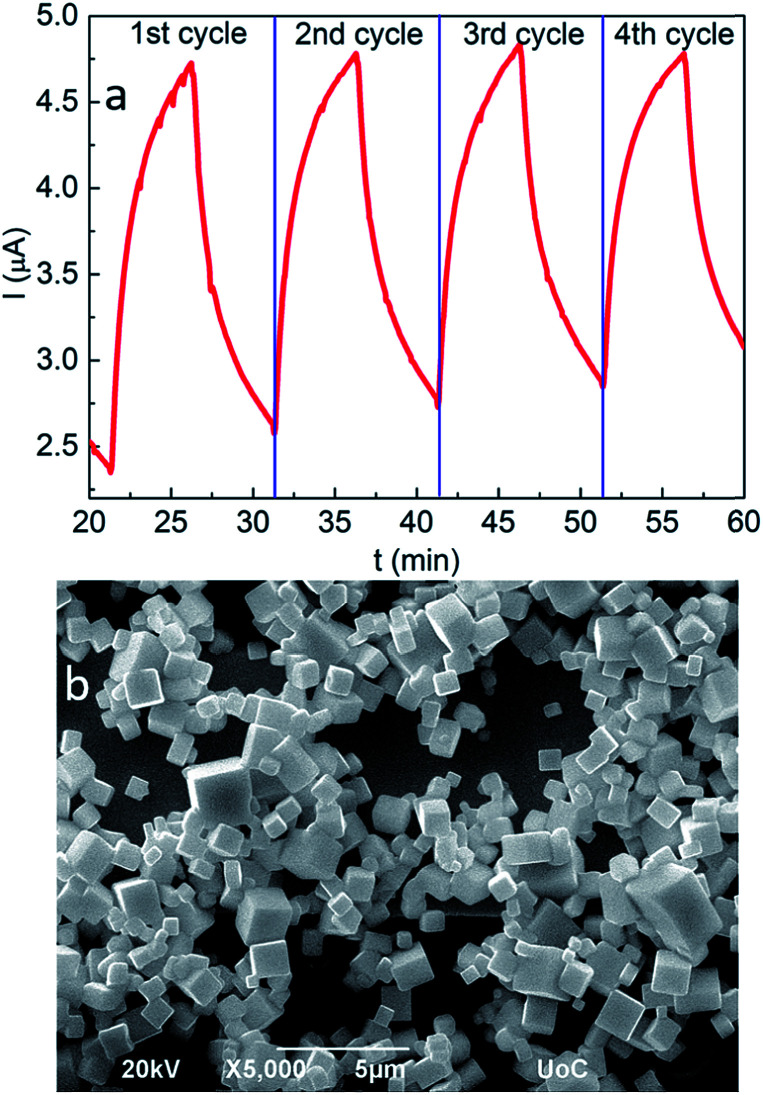
Time dependence of the ozone response upon four successive sensing scans (a) and SEM image of the all-inorganic perovskite nanocubes at the end of the sensing process (b).

**Fig. 5:**
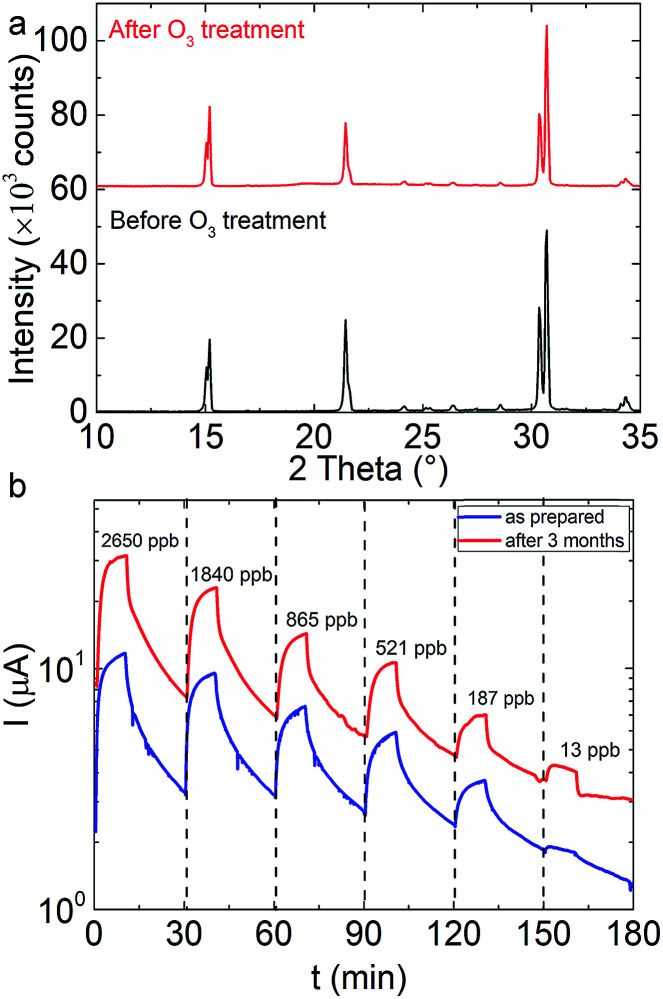
(a) Corresponding XRD patterns of the sensing material before (lower) and three months after the ozone exposure (upper) (images are presented in Fig S3†). (b) Electrical response of an all-inorganic perovskite nanocube sensor as a function of the ozone exposure time at various concentrations in the as-prepared state (blue curve) and that obtained upon the sensor storage for three months under ambient conditions (red curve).

The analysis of the sensing properties in successive ozone/synthetic air switching cycles revealed a remarkable repeatability and stability of the fabricated sensors. In particular, following ozone exposure, the induced current rapidly increases by six times and readily recovers to its initial value upon ozone removal (Fig. 4a). However, it is noted that after a number of successive cycles the current does not fully reverse to its initial value, possibly due to residual adsorbed ozone molecules. O_2_ passivation of surface hole-traps has been observed in nanocrystals of a similar phase.^74,75^ As shown in Fig. 4b, S3b and c†, following a series of four sensing cycles, the nanocubes retain their morphological and stoichiometric features. This can be attributed to the good crystallinity of the nanocubes (Fig. 5a black curve) on the one hand and the fact that no coalescence effects have been observed after ozone treatment, which could degrade the performance of the sensing layer (Fig. 5a red curve).^76^

### Long-term stability

The long-term degradation of the sensing performance has been additionally investigated (Fig. 5b), *via* recording the electrical response of sensor stored for three months under ambient conditions. It is observed that, although the absolute current values are found to be a bit higher, the response and trend are similar to those in the 1st sensing cycle, for all ozone concentrations tested (Fig. 5b). In order to elaborate possible phase changes upon the prolonged exposure to ambient conditions, the XRD pattern of the stored sample is compared to that of the as-prepared one, in Fig. 5a. This comparison indicates that there are no phase alterations or impurity formation. SEM imaging also reveals that the crystals preserve their pristine shape and morphology (Fig. S3†).

### Proposed sensing mechanism

**Fig. 6:**
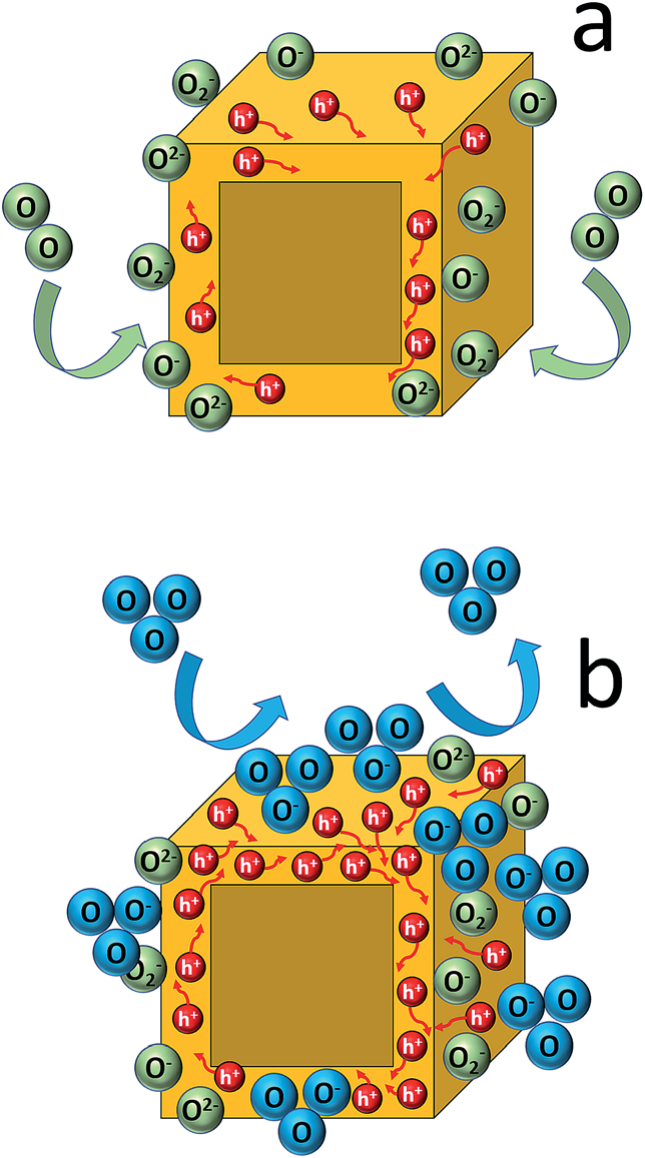
Schematic diagram of the gas sensing mechanism under ambient conditions (a) and after ozone exposure (b).

The absence of any phase transformation and impurity formation, upon exposure to an ozone atmosphere, indicates that the sensing mechanism is different to those reported to date, in which they attribute the sensing effect to a chemical reaction of the perovskite crystals with the gas molecules. For example, molecular dynamics simulations demonstrated that when a perovskite is under a NH_3_ gas environment, the NH_3_ molecule has a tendency to diffuse from the outermost surface layer into the perovskite underlayer leading to distortion of the perovskite lattice.^77^ Moreover, several experimental studies show either an enhancement of the XRD peaks' intensity upon gas exposure, attributed to crystallinity improvement,^78^ or a completely different XRD pattern indicating the formation of a completely new structure.^36,79,80^ In our case such a behaviour is not observed. We can postulate though that our results could be interpreted according to Wolkenstein's model^81^ described below. In particular, when the sensor is exposed to air, adsorbed neutral oxygen molecules (O_2_)_ads_ onto the perovskite surface become partially ionized into O_2_^−^, O^−^ and O^2−^ ions by attracting electrons from the semiconductor valence band. As a consequence, an accumulation layer of holes with lower resistance, covering the whole surface of the nanocubes, is formed due to the reduction in the number of electrons (Fig. 6a). Thus, before the exposure to ozone gas, a core–shell cubic configuration with a resistive core and a conductive hole shell is shaped.^82^ Then, the electrical current is increased due to the adsorption of ozone molecules (electron acceptor), which increases the number of holes in the CsPbBr_3_ perovskite shell lattice. The increasing electron trapping from the valence band of the sensing perovskite element leads to the increase of the current, which is a p-type electrical behaviour (Fig. 6b).^83^ A similar sensing mechanism has been proposed in the case of a photoexcited porous network of the same perovskite phase upon O_2_ exposure.^31^

## C. Experimental

### Materials and methods

#### Chemicals

All reagents were of relatively high-purity. PbBr_2_ (trace metals basis, 99.999%), CsBr (anhydrous, 99.999%), and toluene (anhydrous, 99.8%) were purchased from Aldrich. *N*,*N*-dimethylformamide (DMF, anhydrous, 99.8%) was purchased from Alfa-Aesar. All the reagents were stored and handled under an argon atmosphere in a glove box (MBRAUN, UNILab). Fabrication of nanocubes: in a typical synthesis, a stock solution of commercially available precursors (0.4 mmoles PbBr_2_ and 0.4 mmoles CsBr) in 10 ml of anhydrous DMF was prepared in a sealed vial closed under Ar in a protective atmosphere of a glovebox. The solution was stirred until the dissolution of the precursors. Then a small amount (7.6 μl) of the fresh stock-solution has been deposited on commercial InterDigitated platinum Electrodes (IDEs, 10 μm bands/gaps, DropSens) on a glass substrate and before its evaporation, 7.6 μl of anhydrous toluene was added dropwise to spread on the entire substrate. A thin and uniform liquid layer has been formed. The yellowish color of the solution on the substrate became bright yellow directly after the addition of the toluene, indicating the formation of the crystals. The substrate was left to dry overnight. Well-formed CsPbBr_3_ nanocubes of 500–1000 nm grew on the substrate. The entire process was conducted inside an argon filled glovebox with O_2_ and H_2_O concentrations below 0.1 ppm. Before the ozone sensing, no other treatment has been carried out. The substrates on which the sensors were fabricated were purchased prepatterned from DropSens and were composed of two InterDigitated electrodes with two connection tracks, all made of platinum, on a glass substrate. The space between the interdigitated electrodes was 5 μm.

#### Characterization

The quality of the nanocube films has been examined by the means of Scanning Electron Microscopy (SEM), Energy-Dispersive X-ray Spectroscopy (EDS) and X-Ray Diffractometry (XRD). A SEM (JEOL 7000), equipped with an EDS (INCA PentaFET-x3), was used for the stoichiometric analysis of the samples. XRD studies were performed on a Rigaku D/MAX-2000H rotating anode diffractometer with Cu Kα radiation, equipped with a secondary graphite monochromator. The XRD data at room temperature were collected over a 2*θ* scattering range of 5–90°, with a step of 0.02° and a counting time of 10 s per step. The perovskite covered substrates have been measured without further treatment.

#### Gas sensing measurements

Commercial InterDigitated platinum Electrodes (IDEs, 10 μm bands/gaps, DropSens) on glass substrates were used for direct growth of the all-inorganic metal halide nanocubes for monitoring the electric current variations resulting from their interaction with the ozone gas. The IDEs/glass substrate covered with the nanocubes was placed in a home-made chamber and evacuated down to 10^−3^ mbar. A Keithley 6517A electrometer connected with the IDEs was used to record the electrical current through the nanomaterials, applying a voltage of *V* = 1 volt. The ozone concentration ranged from 2650 down to 4 ppb and was recorded by using an ozone analyzer (Thermo Electron Corporation, Model 49i). Subsequently, a well-defined O_3_ flow of 500 sccm (standard cm^3^ min^−1^) controlled by using a flowmeter was introduced into the chamber for 10 min resulting in an increase of electrical current. The sensor was recovered by introducing synthetic air into the chamber for 20 min with a similar flow, leading to a decrease in electrical current. During the exposure and the recovery processes, the pressure in the chamber was kept constant and equal to 400 mbar. All the experiments were performed at room temperature.

## D. Conclusions

It has been demonstrated that the direct growth of ligand-free, all-inorganic metal halide nanocubes from solution is a convenient preparation method for highly sensitive and durable ozone sensors. The main advantage of this technique is its simplicity as well as the cost-effective fabrication. The sensor is able to work at room temperature, without the need for external stimuli (*i.e.* self-powered), such as UV irradiation or heating and it is fully reversible following the ozone withdrawal. Its sensitivity (54% at 187 ppb ozone concentration) is the highest among those reported in the literature. Furthermore, its sensitivity and durability are by far better than those of the hybrid organic–inorganic metal halide sensing film, which is the only lead halide perovskite material tested for ozone sensing to date, which it lacks stability as it degrades upon exposure to humidity and oxygen. Besides this, the response time is comparable to that of oxides; however, the simplicity of the synthesis process, as well as the use of solely commercially available reactants and solvents, brings about a breakthrough step in gas sensors' development.

This is the first report on the use of all-inorganic metal halide perovskite nanostructures as ozone sensing elements and it will give the pace for further studies of new perovskite materials, such as lead-free compounds, for gas sensing applications.

## Conflicts of interest

There are no conflicts to declare.

## Supplementary Material

NA-001-C9NA00219G-s001
