# Supplementary material for: Ligand-free all-inorganic metal halide nanocubes for fast, ultra-sensitive and self-powered ozone sensors
Source: Nanoscale Adv. 2019 May 22;1(7):2699–706. doi: 10.1039/c9na00219g (PMC9419230; doi:10.1039/c9na00219g)
Supplement: NA-001-C9NA00219G-s001 [file NA-001-C9NA00219G-s001.pdf]

Electronic Supplementary Material (ESI) for Nanoscale Advances.
This journal is © The Royal Society of Chemistry 2019

# -Supporting information-

## Ligand-free all-inorganic metal halide nanocubes for fast, ultra-sensitive and self-powered ozone sensors

K. Brintakis[a], E. Gagaoudakis[a,b], A. Kostopoulou*[a], V. Faka[b,c], A. Argyrou[a,c], V. Binas[a,b,d], G. Kiriakidis[a,b], E. Stratakis*[a,b]

a) Institute of Electronic Structure & Laser (IESL), Foundation for Research and Technology (FORTH) Hellas, P.O. Box 1385, Heraklion 70013, Crete, Greece
b) University of Crete, Department of Physics, 710 03 Heraklion, Crete, Greece
c) University of Crete, Department of Materials Science and Technology, 710 03 Heraklion, Crete, Greece
d) Crete Center for Quantum Complexity and Nanotechnology, Department of Physics, University of Crete, 71003 Heraklion, Greece

Corresponding authors:

- Athanasia Kostopoulou: akosto@iesl.forth.gr,
- Emmanuel Stratakis: stratak@iesl.forth.gr

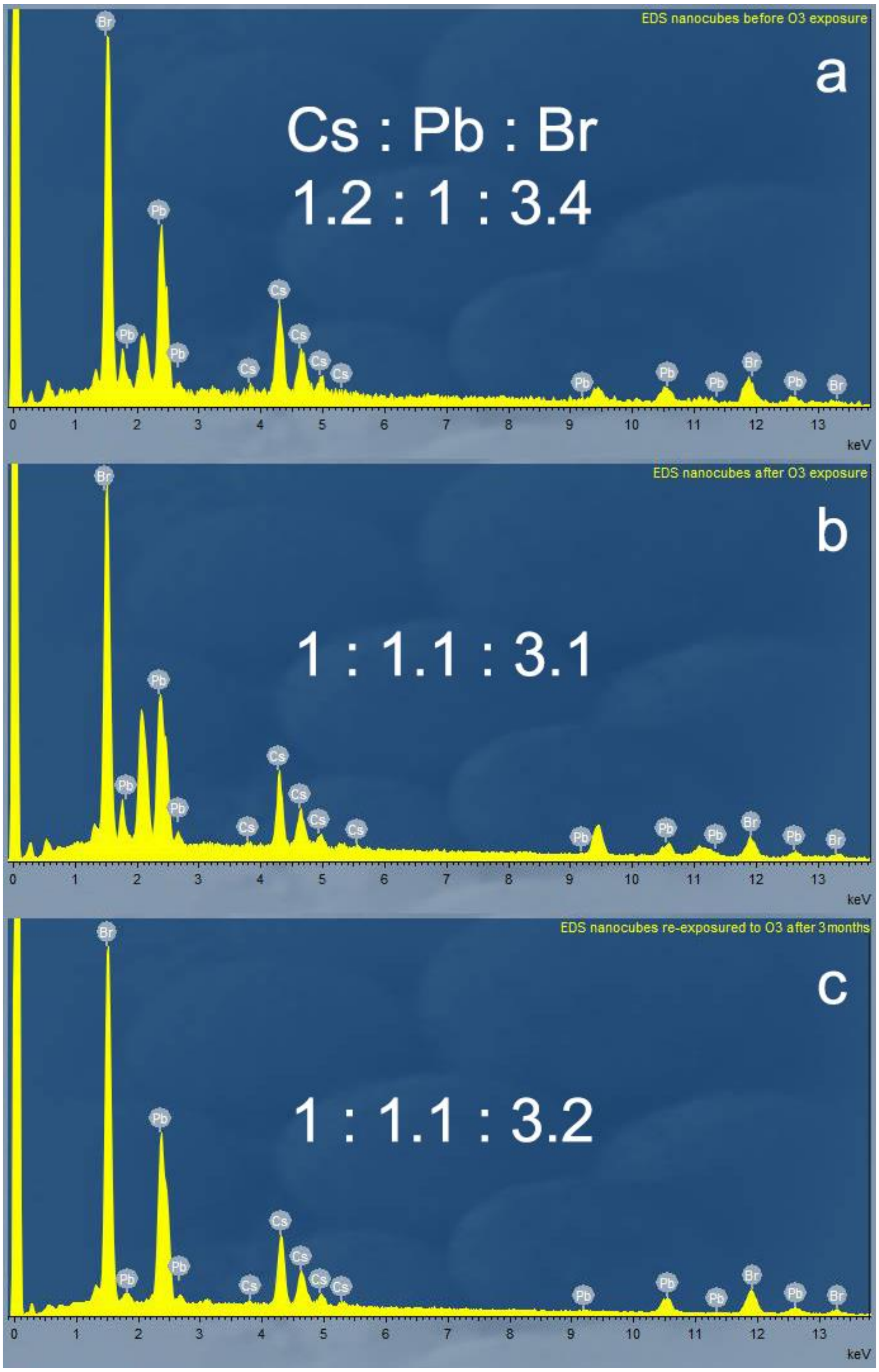


**Figure S1.** SEM-EDS spectra of the $CsPbBr_3$ nanocubes a) before ozone exposure, b) after ozone exposure and c) re-exposure to ozone after 3 months stored at ambient conditions.

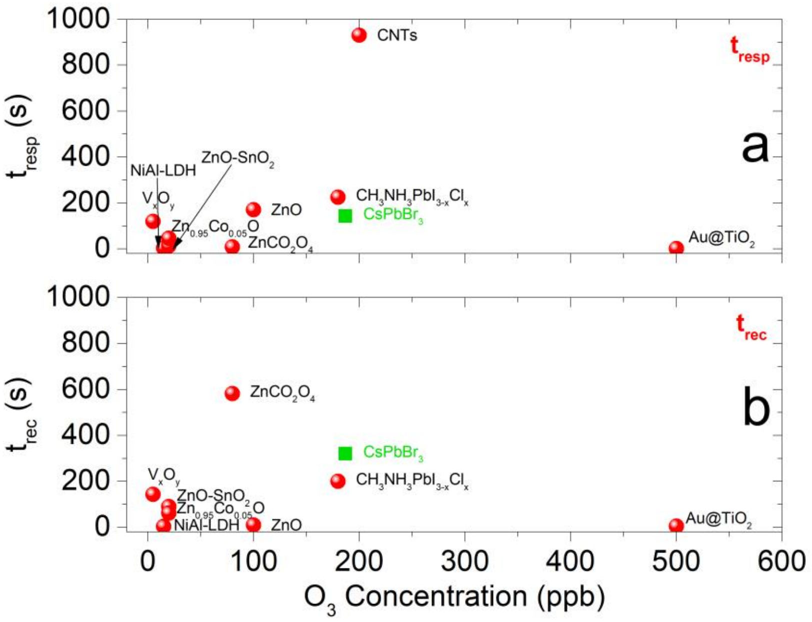


**Figure S2.** Diagrams of response and recovery times of the $CsPbBr_3$ nanocubes compared to the various reported ozone sensing elements. More information for these data is summarized in the table S1.

**Table S1.** Summary of the Phase, morphology, preparation methods, working temperature, sensitivity (S), ozone concentration, t-response : t-recovery times for the ozone sensing elements found in the literature.

| Material | Morphology | Preparation method | $T_{work}$ (°C) | S | $O_3$ conc. (ppb) | $t_{res}$:$t_{rec}$ (s) | Ref. |
|---|---|---|---|---|---|---|---|
| $CsPbBr_3$ | Nanocubes | Solution-based | RT | 54 | 187 | 143:320 | This study |
| $Au@TiO_2$ | Nanoparticles | Sol-gel | RT | 1.1 | 500 | 2:5 | 1 |
| NiAl-LDH | Agglomerated flakes | Hydrothermal | RT | 1.2 | 15 | 4:4 | 2 |
| $CH_3NH_3PbI_{3-x}Cl_x$ | Nanostructured Film | Solution based | RT | 3 | 180 | 225:~200 | 3 |
| $V_xO_y$ | Nanostructured Film | Aerosol Spray Pyrolysis | RT (UV) | 29 | 5 | 120:143 | 4 |
| $TiO_2$-$In_2O_3$ | Film | Impregnation | RT (UV) | 56.5 | 2000 | 115:145 | 5 |
| ZnO-$SnO_2$ | Irregular shaped | Hydrothermal | RT (UV) | 8 | 20 | 13:90 | 6 |
| $Zn_2SnO_4$-RGO | Nanoparticles on rGO flakes | Hydrothermal | 30 | 1.9 | 1000 | tens of min | 7 |
| CNTs | Porous fibrous CNTs | Solution-based | 75 | 2 | 200 | 930:1722 | 8 |
| $ZnCO_2O_4$ | Microspheres | Co-precipitation | 200 | 23.3 | 80 | 8.4:582 | 9 |
| $Zn_{0.95}Co_{0.05}O$ | Thin film | Spray pyrolysis | 250 | 0.4 | 20 | 46:62 | 10 |
| ZnO | Porous nanosheets | Hydrothermal | 300 | 90.5 | 100 | ~170:s | 11 |

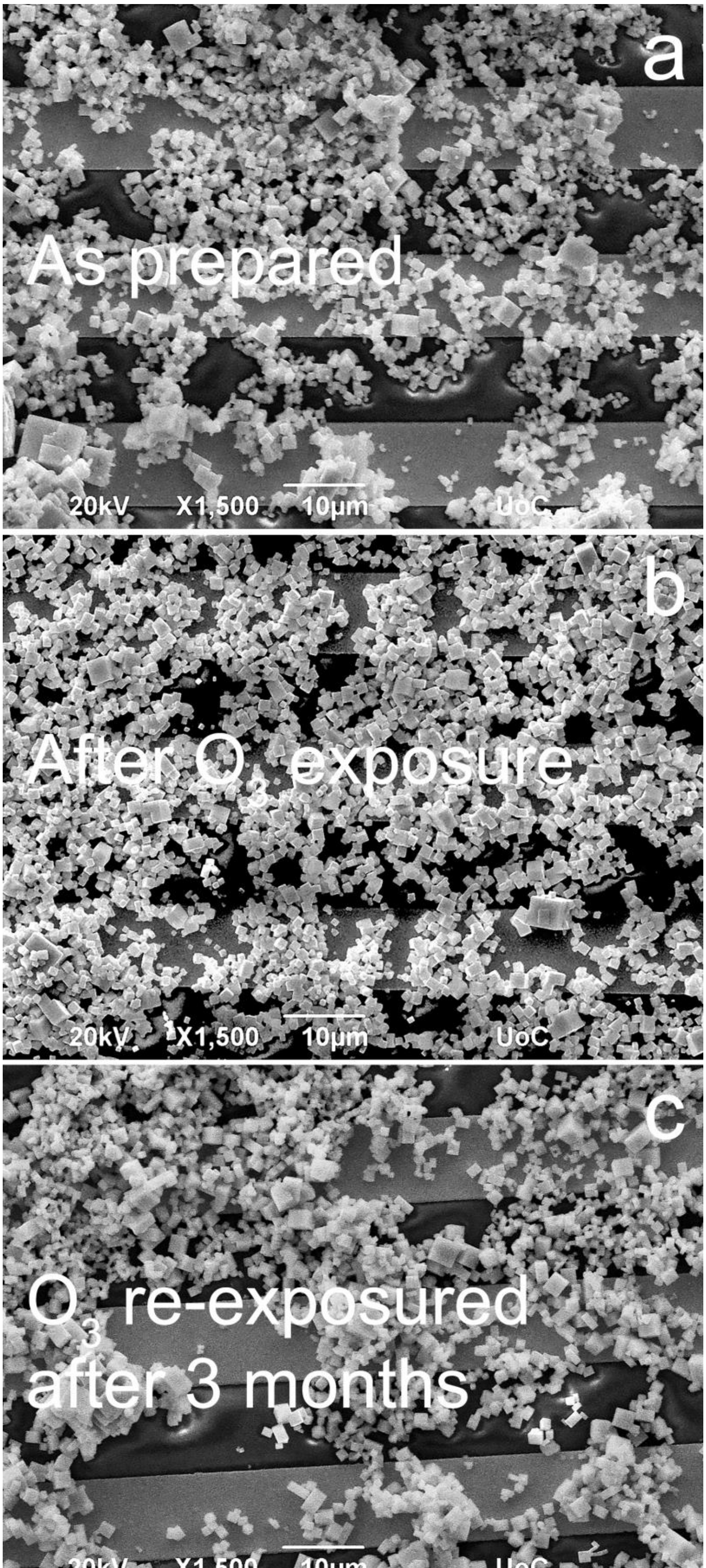


**Figure S3.** SEM images of $CsPbBr_3$ nanocubes a) as prepared, b) after $O_3$ exposure, c) after $O_3$ re-exposure (after 3 months) stored at ambient conditions.

**Reference:**


1 Z. Zhu, J.-L. Chang and R.-J. Wu, *Sensors and Actuators B: Chemical*, 2015, **214**, 56–62.

2 G. Kang, Z. Zhu, B.-H. Tang, C.-H. Wu and R.-J. Wu, *Sensors and Actuators B: Chemical*, 2017, **241**, 1203–1209.

3 G. Kakavelakis, E. Gagaoudakis, K. Petridis, V. Petromichelaki, V. Binas, G. Kiriakidis and E. Kymakis, *ACS Sensors*, 2018, **3**, 135–142.

4 S. Gavalas, E. Gagaoudakis, D. Katerinopoulou, V. Petromichelaki, S. Wight, G. Wotring, E. Aperathitis, G. Kiriakidis and V. Binas, *Materials Science in Semiconductor Processing*, 2019, **89**, 116–120.

5 C.-H. Wu, T.-L. Chou and R.-J. Wu, *Sensors and Actuators B: Chemical*, 2018, **255**, 117–124.

6 L. F. da Silva, J.-C. M'Peko, A. C. Catto, S. Bernardini, V. R. Mastelaro, K. Aguir, C. Ribeiro and E. Longo, *Sensors and Actuators B: Chemical*, 2017, **240**, 573–579.

7 Z. Wang, A. Sackmann, S. Gao, U. Weimar, G. Lu, S. Liu, T. Zhang and N. Barsan, *Sensors and Actuators B: Chemical*, 2019, **285**, 590–600.

8 D. Ziegler, E. Bekyarova, A. Marchisio, J.-M. Tulliani and K. Naishadham, in *2018 IEEE SENSORS*, IEEE, New Delhi, 2018, pp. 1–4.

9 N. Joshi, L. F. da Silva, H. S. Jadhav, F. M. Shimizu, P. H. Suman, J.-C. M'Peko, M. O. Orlandi, J. G. Seo, V. R. Mastelaro and O. N. Oliveira, *Sensors and Actuators B: Chemical*, 2018, **257**, 906–915.

10 Y. J. Onofre, A. C. Catto, S. Bernardini, T. Fiorido, K. Aguir, E. Longo, V. R. Mastelaro, L. F. da Silva and M. P. F. de Godoy, *Applied Surface Science*, 2019, **478**, 347–354.

11 Y.-T. Tsai, S.-J. Chang, I.-T. Tang, Y.-J. Hsiao and L.-W. Ji, *IEEE Sensors Journal*, 2018, **18**, 5559–5565.
